# REMOTE ASYNCHRONOUS FEEDBACK FOR UNSUPERVISED LAPAROSCOPIC TRAINING: THE “LAPP” PLATFORM

**DOI:** 10.1590/0102-672020220002e1712

**Published:** 2023-01-09

**Authors:** Gabriel ULLOA, Andres NEYEM, Gabriel ESCALONA, Catalina ORTIZ, Julian VARAS

**Affiliations:** 1 Pontificia Universidad Católica de Chile, Computer Science Department, School of Engineering – Santiago, Chile;; 2 Pontificia Universidad Católica de Chile, Experimental Surgery and Simulation Center, Department of Digestive Surgery, School of Medicine – Santiago, Chile.

**Keywords:** Laparoscopy, Simulation Training, Surgical Procedures, Operative, Treinamento por Simulação, Laparoscopia, Procedimentos Cirúrgicos do Sistema Digestório

## Abstract

**BACKGROUND:**

The advantages of laparoscopic surgery over traditional open surgery have changed the surgical education paradigm in the past 20 years. Among its benefits are an improvement in clinical outcomes and patient safety, becoming the standard in many surgical procedures. However, it encompasses an additional challenge due to the complexity to achieve the desired competency level. Simulation-based training has emerged as a solution to this problem. However, there is a relative scarcity of experts to provide personalized feedback. Technology-Enhanced Learning could be a valuable aid in personalizing the learning process and overcoming geographic and time-related barriers that otherwise would preclude the training to happen. Currently, various educational digital platforms are available, but none of them is able to successfully provide personalized feedback.

**AIMS:**

The aim of this study was to develop and test a proof of concept of a novel Technology-Enhanced Learning laparoscopic skills platform with personalized remote feedback.

**METHODS:**

The platform “Lapp,” a web and mobile cloud-based solution, is proposed. It consists of a web and mobile application where teachers can evaluate remotely and asynchronously exercises performed by students, adding personalized feedback for trainees to achieve a learning curve wherever and whenever they train. To assess the effectiveness of this platform, two groups of students were compared: 130 participants received in-person feedback and 39 participants received remote asynchronous feedback throughout the application.

**RESULTS:**

The results showed no significant differences regarding competency levels among both groups.

**CONCLUSION:**

A novel Technology-Enhanced Learning strategy consisting of remote asynchronous feedback throughout Lapp facilitates and optimizes learning, solving traditional spatiotemporal limitations.

## RESUMO

**RACIONAL:**

As vantagens da cirurgia laparoscópica sobre a cirurgia aberta tradicional mudaram o paradigma da educação cirúrgica nos últimos 20 anos, tornando-se o padrão em muitos procedimentos cirúrgicos. No entanto, envolve um desafio adicional devido à complexidade para atingir o nível de competência desejado. O treinamento baseado em simulação surgiu como uma solução. No entanto, há uma relativa escassez de especialistas para fornecer *feedback* personalizado. A *Technology Enhanced Learning* pode ser uma ajuda valiosa na personalização do processo de aprendizagem e na superação de barreiras geográficas e temporais que impediriam o treinamento. Atualmente, várias plataformas educacionais estão disponíveis, mas nenhuma delas é capaz de fornecer feedback personalizado.

**OBJETIVOS:**

desenvolver e testar uma prova de conceito de uma nova plataforma de habilidades laparoscópicas da *Technology Enhanced Learning* com feedback remoto personalizado.

**MÉTODOS:**

É proposta a plataforma “Lapp”, uma solução web e móvel baseada em nuvem. É composta por uma aplicação web em que os professores podem avaliar remotamente e de forma assíncrona exercícios realizados pelos alunos, adicionando *feedback* personalizado para os formandos alcançarem uma curva de aprendizagem onde e quando treinam. Para avaliar a eficácia desta plataforma, dois grupos de alunos foram comparados. 130 participantes receberam feedback pessoal e 39 participantes receberam *feedback* remoto assíncrono em todo o aplicativo.

**RESULTADOS:**

Os resultados não mostraram diferenças significativas em relação ao nível de competência entre os dois grupos.

**CONCLUSÕES:**

Uma nova estratégia *Technology Enhanced Learning* que consiste em *feedback* assíncrono remoto em toda a Lapp facilita e otimiza o aprendizado, resolvendo as limitações espaço-temporais tradicionais.

## INTRODUCTION

The advent of laparoscopic surgery has been related to improved clinical outcomes and patient safety, becoming the chosen approach to the vast majority of abdominal surgical procedures^21^. However, complex learning curves associated with higher training costs have created a need for new teaching methods that allow effective training of practical laparoscopic skills^8,13^. Simulation-based training has become a solution to this problem, allowing the trainee to deliberately practice a procedure in a safe and controlled environment with no ethical concerns^1,20^. Studies have shown that direct and personalized feedback is a key element to implementing a successful simulation program^5^. However, experts’ availability to provide feedback has been a critical resource since most tutors are full-time clinicians with several responsibilities, besides mentoring apprentices. Therefore, experts’ relative scarcity to provide direct feedback has been a hurdle to implementing these programs^10^. The use of technology to solve educational problems, also known as Technology-Enhanced Learning (TEL), has emerged as a solution to several learning obstacles, improving traditional classroom settings and using technology as an educational strategy^15^. This allows students to learn at their own pace, acquiring contents that are most relevant to them, customizing the teaching and learning process without geographical or time constraints asynchronously^5,19^.

In healthcare sciences, TEL has made a substantial contribution, being the student’s preferred learning method^3^. In surgical education, the implementation of video libraries as complementary material has a potential benefit to the trainees’ learning process^2^. In laparoscopic surgery, several TEL educational tools have been described; iTrainer, Lap Suturing App, and EcoSurgical have made a substantial contribution to the field, but the lack of direct personalized feedback hinders the ability of these tools to reach the trainees’ maximum potential^6^.

Therefore, the following question arises: Is it possible to create a TEL solution that allows remote asynchronous personalized feedback by experts?

Surgical video assessment in resident training is a recommended educational strategy according to a systematic review. Video review preoperatively and postoperatively seems to enhance residents’ learning processes alongside attendings’ postoperative assessment of the procedure. WebSurg^®^ is one of the platforms available to do so, offering high-quality minimally invasive surgery videos from a variety of settings^12^.

A study conducted by Green and colleagues sought to improve a video platform for learning surgical skills at home^9^. Students watched videos of the instructors completing a set of exercises, to later upload their own video performing the task. In the final stage, peer-review evaluation was available. A satisfaction survey was conducted at the end of the study, and the platform was well evaluated by participants, showing a high level of engagement among students.

Finally, Schmidt and colleagues used a TEL platform to improve laparoscopic suturing and knot-tying techniques in a self-directed manner. Two groups were randomized to either first-person perspective (a combination of endoscopic view plus view of hands/instruments motion) or endoscopic view only^17^. Their results indicate that the first-person perspective does not improve performance, but it is a feasible and well-accepted training method.

All these studies indicate that self-directed learning using technology is possible and may even provide better results than the in-person synchronic approach. However, there are a variety of learning experiences depending on the availability of expert feedback. Some platforms focus solely on providing access to audiovisual material, while others only provide feedback based on examination scores. In addition, most platforms focus on assessing theoretical knowledge without providing the experiential learning experience that is crucial in surgical training. To solve these problems, a cloud-based web and mobile application called “Lapp” is proposed, allowing direct interaction between trainee and expert through a remote asynchronous mentoring approach, providing remote personalized feedback, and enhancing the educational process.

## METHODS

A variety of healthcare professionals were recruited, including general surgeons, gynecologists, veterinarians, and a pediatric surgeon. A basic laparoscopic skills training course was designed, consisting of 10 exercises that were incremental in difficulty. The exercises included bean transfer, cutting patterns, and performing silicone sutures. All students faced the same challenges, but some of them received the initial instructions and feedback through Lapp, while others received it in person in a simulation center.

For this, we designed a platform with several audiovisual tools to facilitate remote deferred feedback, which has proved to enhance the learning process^14,22^. A lean startup methodology, which consists of constant improvement through users’ feedback and iterations, was used to design, implement, and test this tool^7^.

### Lapp Design

**Figure 1.**
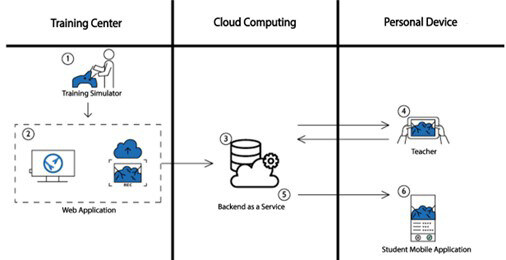
Sequence diagram showing the flow of communication between the different components of the platform.

Lapp is a user-centered mobile cloud computing platform that enables experiential learning of laparoscopic surgery’s practical skills. It is based on high-quality audiovisual material that demonstrates the step-by-step execution of different exercises. Students watch the tutorials and then record themselves performing the procedure on their own. The videos are uploaded to the platform in order to be reviewed by experts. Through various audiovisual interactive tools, tutors provide personalized remote asynchronous feedback until the expected competency level is achieved. It is foreseen that the student will improve at least as much as with a tutor by their side (see Figure 1).

### Platform Architecture

**Figure 2.**
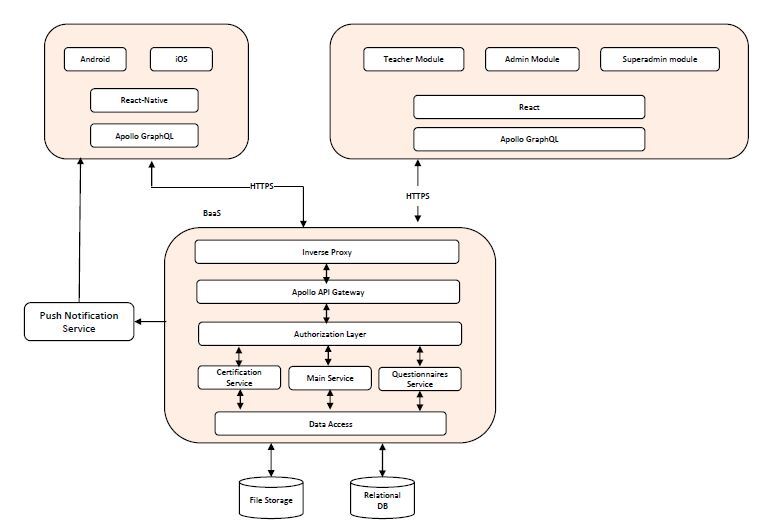
General architecture for the proposed user-centered mobile cloud computing platform.

The Lapp architecture is based on a mobile cloud computing strategy that involves outsourcing part of the processing load to the cloud and getting back the results^16^. The advantage of this approach is to overcome limitations like high consumption processes, data persistence across time, different students’ contexts, and spatiotemporal limitations^4^. A full rundown of the architecture is shown in Figure 2.

### Mobile App

**Figure 3.**
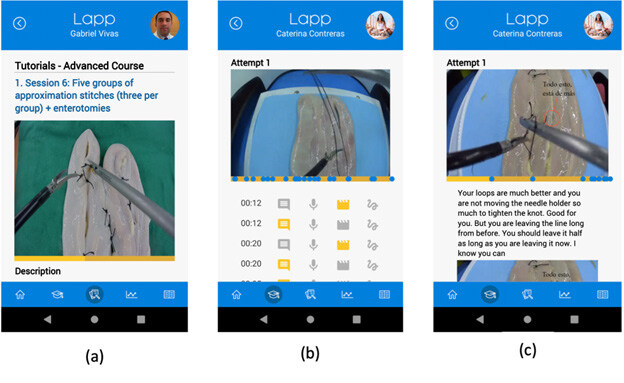
Mobile application. (a) Tutorial screen, where the students watch tutorial videos of the exercises to perform; (b) Evaluation screen, where the students watch the feedback provided by the teacher; (c) Evaluation screen, with a drawing over the image to mark a correction.

The mobile app increases the availability of educational content by providing a “ubiquitous learning” environment, allowing the student to access high-level educational materials and train at any time and place, using a mobile device in a user-friendly manner. To create the app, React Native, a native scripting framework that creates native apps with an interface to process JavaScript code at the time of execution, was chosen (Figure 3).

### Website

**Figure 4.**
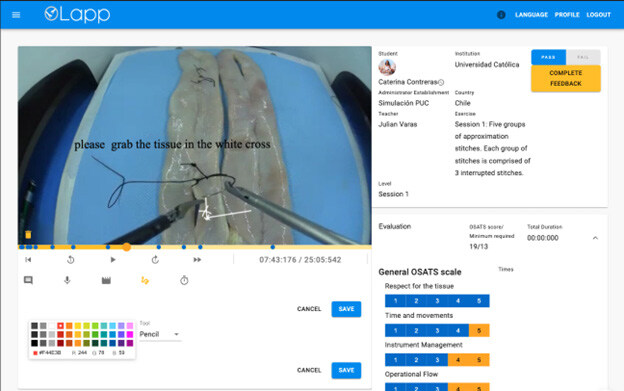
Web application. As shown in the screen, the teacher reviews an evaluation. Several audiovisual tools are available to facilitate the evaluation and provide multimodal feedback.

The website is the main user component of the platform. Teachers and administrators will use it to upload training videos and other educational materials and view progress statistics (see Figure 4). The Web App was developed using React, which shares the same base as React Native (and therefore allows reusing the code). It possesses an advanced performance level due to a “Virtual Dom” that uses JavaScript to calculate the interphases’ state and determine when visualizations should be modified^11^.

### Cloud Backend as a Service (CBaaS)

This component is in charge of processing and storing the data. To do so, both the previously described components interact with CBaaS through HTTPS requests for the secure and precise transfer of data. An advantage of this interface is implementing a microservice architecture and allowing the logical separation of components into independent pieces, being more scalable and maintainable than a monolith arquitecture^18^.

Students will access the platform in independent temporal spaces, meaning the system must ensure centralized access alongside consistency in information storage. To do so, Amazon Web Service (AWS) systems are used to hold audiovisual content and all the information related to student evaluations.

### Training Methods

Participants were randomly assigned to one of the following two groups: in-person synchronous feedback (group A) and remote asynchronous feedback through Lapp (group B). The passing criteria were based on the time to complete the procedure and technical competency (Table 1). Participants were able to revise the tutorials whenever they deemed necessary.

**Table 1.** The passing criteria to complete the procedure and technical competency.

	Approval time (s)
Bean drop	24
Row passing	28
Checkboard	68
Peg transfer	16
Silicone suture	17
Interrupted intracorporeal silicone suture	90
Continuous intracorporeal silicone suture	270
Pattern cutting	98
Endoloop	53
Laparoscopic cannulation	65

In addition, a user experience survey was conducted at the end of the training period among the remote asynchronous feedback group of participants. It consisted of 14 Likert-scale questions regarding the usability of the platform. The survey was conducted online throughout the Lapp platform.

### Statistical Analysis

Nonparametric tests were used. The Wilcoxon test was applied to assess the progression between each participant’s score before and after the training. The Mann-Whitney U test was used to evaluate the differences between both groups. Descriptive statistics were also applied to characterize the data.

The study was approved by the Ethics Committee of the Pontificia Universidad Católica de Chile (number 170518000).

## RESULTS

A total of 420 participants were recruited. In all, 288 participants were assigned to the in-person feedback (group A) and 132 to the remote asynchronous feedback (group B).

Notably, 169 participants were able to successfully complete the training course, with 130 participants being from group A and 39 from group B. All of them met the approval criteria, with statistically significant differences (p<0.001) in the improvements between the first registered score and the passing score for every participant in each group (Table 2).

**Table 2.** Groups A and B of participants.

Laparoscopic cannulation	Endoloop	Pattern cutting	Continuous intracorporeal silicone suture	Interrupted intracorporeal silicone suture	Silicone suture	Peg transfer	Checkboard	Row passing	Bean drop	
89.5 (20.0–327.0)	54 (20.0–184.0)	136.5 (60.0–409.0)	453.5 (210.0–1500.0)	181.5 (65.0–494.0)	75 (25.0–243.0)	46.5 (16.0–154–0)	127 (49.0–288.0)	45.5 (27.0– 115.0)	61.5 (25.0–183.0)	First score group A
39 (12.0–123.0)	38 (18.5–104.6)	125 (75.0–394.8)	323.4 (1.6–754.5)	101.8 (58.0–415.0)	66.6 (15.9–187.6)	41 (17.0–81.6)	102 (46.0–193.9)	41.3 (16.8–86.0)	45 (21.6–112.0)	First score group B
34 (15.0–62.0)	31 (13.0–53.0)	73 (42.0–96.0)	217 (165.0–270.0)	71 (46.0–90.0)	15 (11.0–17.0)	14 (11.0–16.0)	53 (32.0–68.0)	20 (11.0–27.0)	20 (15.0–24.0)	Approval score group A
29 (11.0–54.0)	30 (16.2–41.2)	80 (52.0–93.0)	223.1 (133.0–267.0)	68 (52.0–85.0)	15.2 (10.7–17.0)	14 (11.0–16.0)	55 (35.0–63.0)	23 (15.0–27.0)	20 (15.0–24.0)	Approval score group B
**p<0.01**	**p<0.01**	**p<0.01**	**p<0.01**	**p<0.01**	**p<0.01**	**p<0.01**	**p<0.01**	**p<0.01**	**p<0.01**	Group A: first vs. approval
**p<0.01**	**p<0.01**	**p<0.01**	**p<0.01**	**p<0.01**	**p<0.01**	**p<0.01**	**p<0.01**	**p<0.01**	**p<0.01**	Group B: first vs. approval

At the end of the training period, 32 (80%) participants from group B completed the user experience survey. As can be seen in Table 3, most participants had a positive opinion about the platform. However, some participants reported problems regarding navigation on the platform.

**Table 3.** Opinions about the platform.

	P25	Median	P75
I find the platform easy to use.	2	4	4.25
I feel comfortable with the time spent navigating the platform.	3	4	5
I am satisfied with the instructions and assistance information.	4	4	5
It is easy to navigate through the app.	3	4	5
The app is pleasant.	3	4	5
The app looks simple and clean.	4	4	5
I would like to use this platform frequently.	3	4	5
I find the platform unnecessary complicated.	1	2	2
I think I would require further assistance to be able to use the platform.	1	2	3
I believe all platform functions are well integrated.	2.75	4	4
I believe the platform was inconsistent.	2	2	3.25
I believe most people would learn how to use the platform very fast.	4	4	4
I find the platform to be uncomfortable.	2	2	4
I felt safe using the platform.	3	4	5

P25: pass 25%; P75: pass 75%.

## DISCUSSION

Appropriate feedback is an essential component of the learning process, being particularly critical in the acquisition of practical skills. However, experts are often clinicians with several responsibilities in addition to their role as educators, which have become specifically notorious during the pandemic. As COVID-19 has taken an unprecedented toll in healthcare systems and an “all hands-on-deck” approach has been required, the relative scarcity of tutors to provide high-quality personalized feedback has reached a precarious level. In this scenario, there is an urgent need to provide TEL solutions that facilitate learning in all contexts, making asynchronous personalized feedback a reality.

The Lapp is an educational software platform to enhance laparoscopic skills in surgical education. This type of TEL plays a vital role in supporting several aspects of teaching laparoscopic surgery by enabling students to train and gain laparoscopic skills while receiving direct personalized feedback in an asynchronous fashion.

The proposed architecture is based on a mobile cloud computing strategy. This approach has advantages that facilitate remote asynchronous feedback. A cloud environment allows processing and storage of data related to training sessions and evaluations, i.e., data can be stored for long periods of time and easily accessed through the mobile application and web page, thus allowing to overcome traditional spatiotemporal limitations and providing an ubiquitous learning experience.

This study showed that participants significantly improved their skills after training with Lapp. Moreover, all participants who finished the course met the approval criteria. When comparing the approval scores obtained by both groups, there were no significant differences in most modules.

Regarding time to complete the procedure, there is more homogeneity in participants who trained using Lapp, indicating that even though no significant differences were found in procedural times among both the groups, the participants who trained with Lapp achieved a more similar level of competency.

In addition, most participants found Lapp to be a user-friendly platform, not adding extra difficulties to the learning process. However, there is room for improvement so that all participants feel comfortable using the platform.

It is relevant to address the fact that despite the positive results presented, 54% of participants from group A and 70% of participants from group B were not able to complete the procedure. This elevated drop-out rate should be embraced in future studies.

## CONCLUSIONS

A novel TEL software that allows remote asynchronous feedback is effective for the acquisition of practical skills, facilitating learning while solving spatiotemporal limitations that otherwise would preclude the training to happen.
